# Improper activation of D1 and D2 receptors leads to excess noise in prefrontal cortex

**DOI:** 10.3389/fncom.2015.00031

**Published:** 2015-03-11

**Authors:** Michael C. Avery, Jeffrey L. Krichmar

**Affiliations:** ^1^Systems Neurobiology Laboratory, Salk Institute for Biological StudiesSan Diego, CA, USA; ^2^Department of Cognitive Sciences, University of CaliforniaIrvine, CA, USA; ^3^Department of Computer Sciences, University of CaliforniaIrvine, CA, USA

**Keywords:** dopamine, schizophrenia, computational modeling, D1 receptor, D2 receptor

## Abstract

The dopaminergic system has been shown to control the amount of noise in the prefrontal cortex (PFC) and likely plays an important role in working memory and the pathophysiology of schizophrenia. We developed a model that takes into account the known receptor distributions of D1 and D2 receptors, the changes these receptors have on neuron response properties, as well as identified circuitry involved in working memory. Our model suggests that D1 receptor under-stimulation in supragranular layers gates internal noise into the PFC leading to cognitive symptoms as has been proposed in attention disorders, while D2 over-stimulation gates noise into the PFC by over-activation of cortico-striatal projecting neurons in infragranular layers. We apply this model in the context of a memory-guided saccade paradigm and show deficits similar to those observed in schizophrenic patients. We also show set-shifting impairments similar to those observed in rodents with D1 and D2 receptor manipulations. We discuss how the introduction of noise through changes in D1 and D2 receptor activation may account for many of the symptoms of schizophrenia depending on where this dysfunction occurs in the PFC.

## Introduction

Schizophrenia is a debilitating mental disorder that compromises normal perceptual processes. It is widely known for its ability to cause sensory hallucinations and delusions, so called “positive symptoms.” Negative symptoms, such as apathy and emotional withdrawal, and cognitive symptoms, such as poor attention and working memory, are also common in patients diagnosed with schizophrenia and are often left untreated in schizophrenic patients (Miyamoto et al., 2012).

Abnormalities of the dopaminergic system in the prefrontal cortex (PFC) have been associated with the pathophysiology of schizophrenia (Weinberger, 1987; Goldman-Rakic, 1999; Winterer and Weinberger, 2004; Durstewitz and Seamans, 2008). Specifically, psychosis can result from drugs such as amphetamines and cocaine, which enhance dopamine release in PFC (Curran et al., 2004). Antipsychotic medications, including haloperidol and chlorpromazine, have been shown to influence the dopaminergic system primarily by antagonizing D2 receptor sites (Seeman, 1987; Lidow et al., 1998a). Genetic variations in dopaminergic genes such as catechol-O-methyltransferase (COMT), which breaks down dopamine after release, have also been linked to schizophrenia (Weinberger et al., 2001; Winterer and Weinberger, 2004).

Several models have been developed that shed light on the underlying mechanisms that give rise to symptoms of schizophrenia (Rolls et al., 2008; Murray et al., 2014). Connectionist models suggest that cognitive symptoms of schizophrenia occur as a result dopaminergic neurons changing the gain of activity in working memory (Cohen and Servan-Schreiber, 1992; Braver et al., 1999). The dynamical systems hypotheses, on the other hand, suggest that instabilities in cortical attractor states can give rise to symptoms of schizophrenia (Loh et al., 2007; Durstewitz and Seamans, 2008). An imbalance in D1/D2 receptor activation, which causes changes in NMDA and GABA conductances, is thought to be the mechanism underlying these changes.

Although these models can account for many symptoms associated with schizophrenia, they lack important details at the microcircuit level regarding D1 and D2 receptor distributions and the changes that these receptors have on the response properties of neurons within the PFC circuit (Wang et al., 2004; Santana et al., 2009; Noudoost and Moore, 2011; Gee et al., 2012; Puig and Miller, 2014). We suggest that weak-activation of D1 receptors and over-activation of D2 receptors introduces noise into the frontal cortices through different routes. Weak activation of D1 receptors in supragranular layers introduces noise between PFC columns, which could potentially lead to cognitive symptoms. Noise in this context is excess cortico-cortical excitatory input within the PFC. Over-activation of D2 receptors in infragranular layers also leads to noise in the PFC, however, through cortico-striatal connections.

Using computational modeling as a mechanistic description for how D1/D2 distributions lead to internal and external noise in the PFC, we were able to match human data showing behavioral deficits for schizophrenic patients on oculomotor delayed response tasks, as well as rodent empirical data showing influences on set-shifting with D1/D2 receptor manipulations (Ragozzino, 2002; Floresco et al., 2006; Nikiforuk, 2012). Though we implement this as an impairment in a specific region (dlPFC) for a specific task (ODR task), we suggest that similar distributions of D1 and D2 receptors would likely be disrupted throughout the PFC (Goldman-Rakic et al., 1990), and that this circuit dysfunction is highly stereotyped and would ultimately be the root of cognitive, positive, and negative symptoms of schizophrenia depending on the input and output regions of the affected PFC area.

## Methods

**Figure 1:**
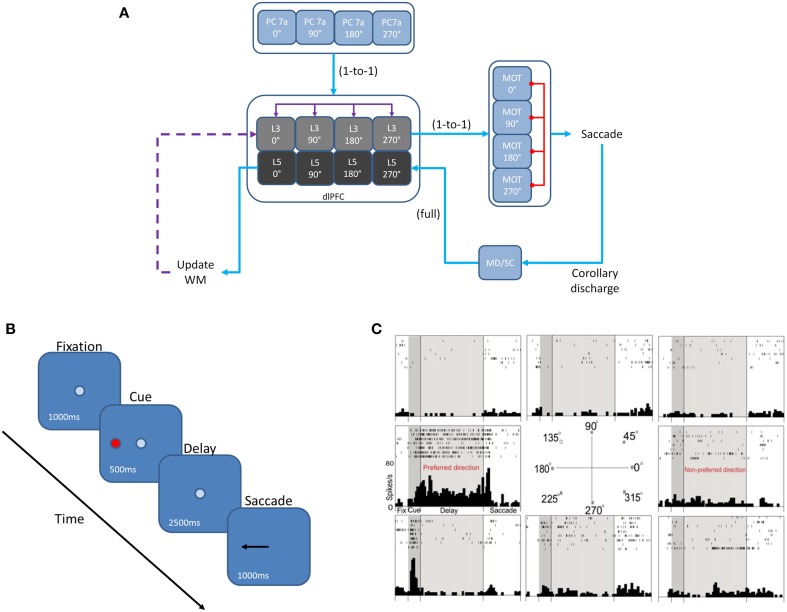
**Network architecture, experiment, and neural responses. (A)** The model contained 4 input areas (PC 7a), each with a preferred saccade direction, which projected topographically to layer 3 of four cortical columns (that is, PC neurons coding for 180° projected to layer 3 neurons coding for 180°). The layer 3 neurons also outputted topographically to motor output areas in order to bias motor responses. Layer 5 neurons in each cortical layer received input from the MD/SC in a non-topographic manner. These neurons, in turn, projected to a set of layers involved in updating working memory. This unit was composed of a non-specific inhibitory layer, whose function was to clear working memory after a behavioral response was made, as well as modeled basal ganglia, which disinhibited a thalamic layer and allowed new information to be gated into the cortex via excitatory projections. Red arrows are inhibitory, blue arrows are excitatory, and purple arrows are excitatory + inhibitory. **(B)** We modeled our experiment after the oculomotor delayed response (ODR) behavioral paradigm. This task is broken down into four stages: fixation, cue, delay, and response. The subject must fixate on a visual screen until a cue is briefly presented. After the cue is flashed there is a delay period (2.5 s in our model) during which the subject must remember where the cue was. Lastly, the subject must saccade to the place on the screen where the subject thought the cue was presented. **(C)** Typical response of a recorded PFC neuron in the ODR task. In this case, the neuron showed persistent activity when a cue is presented at 180°. This is considered the neurons “preferred direction.” This neuron is non-responsive to cues at other spatial locations (non-preferred directions) (adapted from Wang et al., 2007).

We developed a spiking neural network model that included a dlPFC with four-two layer columns each with a preferred saccade direction, a parietal cortex, basal ganglia, superior colliculus, and four motor output areas (Figure 1A). In addition, the model incorporated dopaminergic neuromodulation, including simulated D1 and D2 receptors. This is similar to a recent model we developed that included D1, α2A, and α1 receptors (Avery et al., 2013). We tested our model on the oculomotor delay response (ODR) task, in which a subject must remember the location of a briefly flashed cue over a delay period then saccade to that location (Figure 1B). Figure 1C shows the firing rate of a PFC neuron that was recorded during an ODR task (Wang et al., 2007). The neuron showed persistent firing during the delay period when the cue was presented at 180°. This is considered the “preferred direction” for this neuron. If the cue was presented at any other spatial location (non-preferred direction), the neuron would not show persistent firing during the delay. Our goal was to build a model that could explain the symptoms of schizophrenia, but that took into account the different distributions and cellular affects that are currently known for D1 and D2 receptors. To develop our model, we used a publicly available simulator, which has been shown to simulate large-scale spiking neural networks efficiently and flexibly (Richert et al., 2011). The total simulation time of the experiment was 5 min. This took approximately 37 min to run on an NVIDIA Tesla M2090 GPU with 6 GB of global memory, 512 cores (each operating at 1.30 GHz) grouped into 16 SMs (32 SPs per SM), and a single precision compute power of 1331.2 GFLOPS.

### Network model

**Figure 2:**
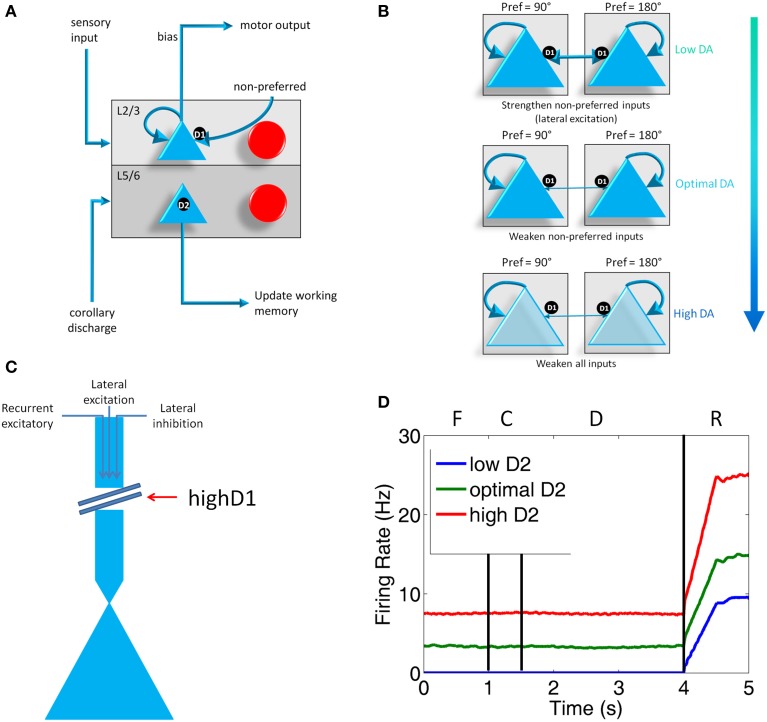
**Individual column architecture and neuromodulatory effects. (A)** Within a column in the PFC, neuromodulators were modeled by changing the strength of inputs from non-preferred directions (D1 receptors) between layer 2/3 neurons in different columns and the strength layer 5 neuronal responses (D2 receptors). As in Figure 1, this architecture also shows how layer 5 neurons in each column received input from the MD/SC and output to a non-specific inhibitory group and the basal ganglia in order to clear working memory and update other columns, respectively. **(B)** Effects of dopamine receptor D1 on layer 3 neurons in the columns of the model. When DA is low (top), connections between columns (non-preferred excitatory inputs) are enhanced, which leads to degradation in spatial tuning. At optimal levels of DA, non-preferred inputs are blocked from other columns, which enhances spatial tuning with the working memory circuits. When DA is high, D1 weakens all inputs to neurons in layer 3 of the cortical columns. **(C)** This figure illustrates the effects that high D1 receptor activation has on our model. High D1 stimulation blocks all inputs to layer 3 neurons, including recurrent excitatory inputs within a column, lateral excitatory inputs from other columns, and lateral inhibitory inputs from other columns. It should be noted that, even though this is how we implemented this functionally in our model to match physiological data, the details of this mechanism have not been completely resolved experimentally. **(D)** Plot showing what layer 5 neuron firing rates look like when D2 receptor stimulation is low, optimal, and high during the Fixation (F), Cue (C), Delay (D), and Response (R) phases of the task. Firing rates were smoothed using a moving average.

**Figure 3:**
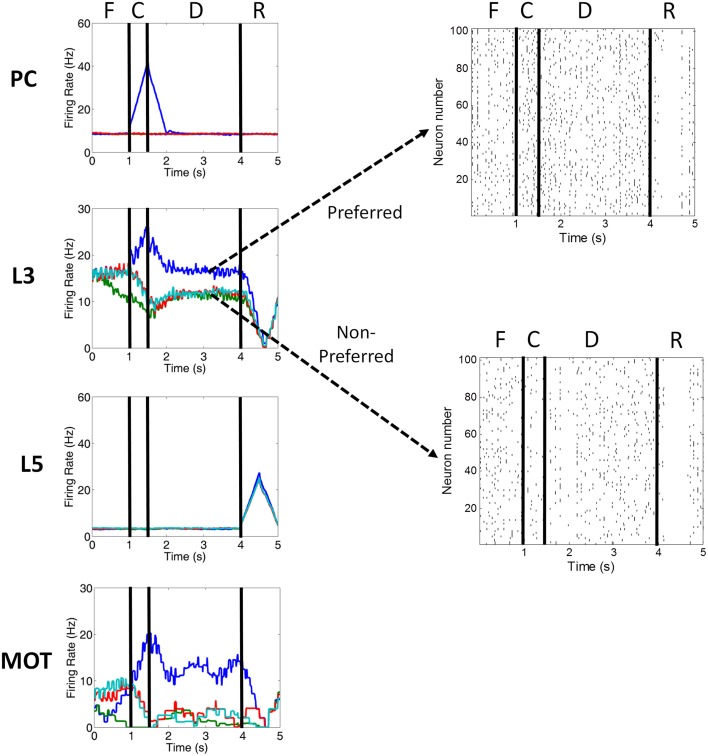
**Firing rate activity of neurons in the PC, PFC L3, PFC L5, and MOT for a single trial**. Typical firing rate activity (smoothed and averaged) of PC, PFC layer 3, PFC layer 5, and MOT neurons during a single working memory trial when DA levels were optimal. PC neurons encoding the preferred direction (blue) are briefly activated when the cue is presented. Layer 3 neurons then hold onto this direction in working memory and drive neurons in the motor response layer, MOT. Layer 5 neurons, on the other hand, fire during the response phase of the task due to a corollary discharge mediated by the MD/SC and clear working memory in layer 3. Fixation (F), cue (C), delay (D), and response (R) periods are indicated at the top. Raster plots for preferred and non-preferred directions are shown in the right. Red, teal and green are non-preferred directions.

The dlPFC portion of the model contained four, two layer cortical columns representing visuospatial working memory circuits, in which each column had a preferred saccade direction of 0, 90, 180, or 270° (Figure 1A). The two layers make up the deep supragranular (layer 3) and upper infragranular (layer 5) layers. Our current understanding of the microcircuitry of the dlPFC suggests that the supragranular layers are where working memory related activity takes place and the infragranular layers are where response-related activity takes place (Arnsten et al., 2012). The supragranular layers of each of the four columns receive visual input from four different parietal cortex (PC 7a) layers and from lateral excitatory and inhibitory connections within the PFC as shown by the purple arrows in Figure 1A (Goldman-Rakic, 1995). These neurons fire in response to the stimulus, hold delay related activity in working memory, and are modulated by D1 receptors (Figures 2A, 3). Each supragranular layer in a column is also involved in biasing motor outputs through projections to four motor (MOT) areas, which accumulate evidence in order to make a saccade direction decision (Schall et al., 2011). Lateral inhibition between MOT neurons was added to promote competition.

Figures 1A, 2A show that infragranular layers receive subcortical inputs from the superior colliculus via the mediodorsal thalamus (MD/SC layer) (Stepniewska and Kosmal, 1986; Sommer and Wurtz, 2006). The SC → MD → PFC pathway has been studied in detail and it has been suggested that the response in infragranular layers is from a corollary discharge that takes place after an eye movement and acts as an efference copy of the motor movement (Wang et al., 2004; Sommer and Wurtz, 2008). The corollary discharge in our model was simulated by briefly driving MD/SC neurons with Poissonian spike inputs at 35 Hz for 500 ms at the beginning of the response phase (4 s into the trial). MD/SC neurons drove infragranular (layer 5) neurons in all cortical columns as can be seen in the L5 firing rate plot in Figure 3.

L5 neurons, in turn, output to the BG (Arnsten, 2011), which disinhibits the thalamus, and to an “inhibitory pool” of neurons, which project to L2/3 excitatory neurons. This is designated in Figures 1A, 2A as the box labeled “Update Working Memory.” L5 projections to the inhibitory pool act to clear working memory. We remain agnostic to the exact circuit involved in this clearing of working memory, however, it could involve either cortico-cortical or cortico-striatal routes. We implemented this as a non-specific projection from the thalamus to all columns. This, however, is an assumption of the model. It is possible that the projections from the thalamus to PFC are explicit and allow for highly specific gating of information in working memory. The clearing of working memory can be seen in the dip in firing rates of L3 neurons during the response phase in Figure 3. L5 output to the BG releases inhibitory control over excitatory thalamic inputs project to other columns. This “cortico-striatal loop,” which acts as a means for updating working memory, has been suggested by Frank and colleagues (Frank et al., 2001; Frank, 2011) and has been experimentally supported (Baier et al., 2010; Voytek and Knight, 2010). Frank and colleagues, however, suggest that the updating of working memory is mediated by D1-expressing neurons in the striatum (“Go pathway”) and the maintenance of working memory is mediated by D2-expressing neurons (“No-go pathway”).

To construct our model, we used a publicly available simulator (http://www.socsci.uci.edu/~jkrichma/CARLsim/), which has been shown to simulate large-scale spiking neural networks efficiently and flexibly (Richert et al., 2011). The model contained a total of 57,212 neurons and approximately 30 million synapses. Connection probabilities in our cortical column model, which were adapted from Wagatsuma et al. (2011), can be found in Table 1. The number of neurons in each area is shown in Table 2. Within each column layer, there are excitatory-excitatory, excitatory-inhibitory, inhibitory-excitatory, and inhibitory-inhibitory connections (not shown in Figure 2A). There are no connections existing between layer 3 and layer 5 neurons within a column. All other connection probabilities between neural groups were set equal to 0.1. The simulation consisted of 50 trials at 6 s per trial.

**Table 1:**
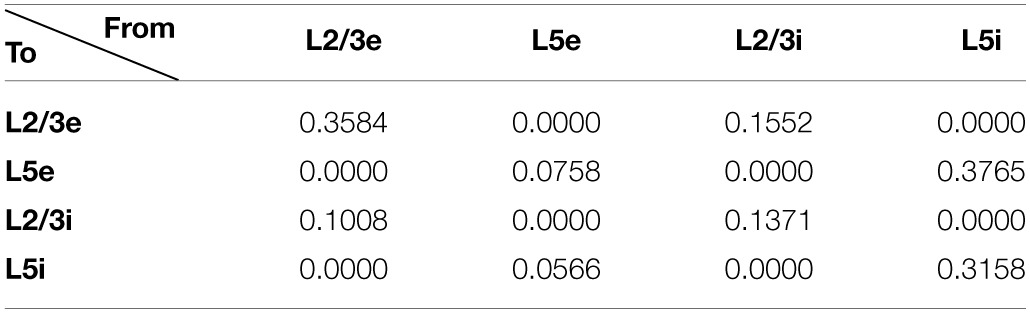
**Cortical connection probabilities within a column**.

**Table 2:** **Number of neurons in each area of the network**.

**Neural area**	**Excitatory neurons**	**Inhibitory neurons**
**SUBCORTICAL**		
BG	–	1000
MD/SC	1000	–
VTA (dopamine)	1000	–
**CORTICAL COLUMN**		
Layer 2/3	2585	729
Layer 5	606	133
**OTHER CORTICAL**		
PC	1000	–
Motor	1000	–

**Figure 4:**
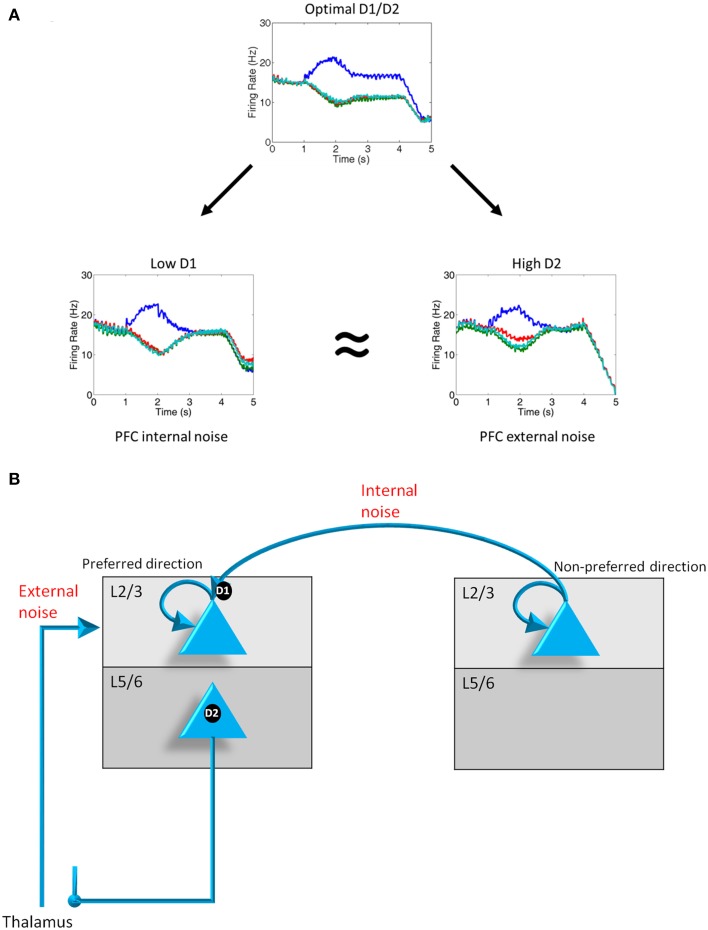
**D1, D2 and internal and external noise. (A)** Plots showing the average firing rate from 1000 neurons on a representative trial when D1 and D2 levels are optimal, D1 levels are low, and D2 levels are high. When D1 and D2 levels decrease and increase, respectively, noise is introduced into the system as can be in the converging of activity of all 4 groups shown from *t* = 2 to *t* = 4 s. Noise as a result of D1 receptor under-stimulation can be thought of as “internal” noise since it is caused by an increase in the strength of excitatory connections between columns with the PFC. Noise resulting from D2 receptor over-activating can be thought of as “external” noise since it is caused by a gating in of thalamic input to layer 2/3 neuron in the PFC. **(B)** Diagram demonstrating the differences in the source of noise that is attributed to D1 under-activation and D2 over-activation.

As in any large-scale network model, tuning the parameters can take a considerable amount of time and effort. As a whole, the network was first tuned for the “optimal” state (see Figure 4A). This was achieved by initially tuning a single layer 2/3 column to have a bi-stable state of persistent and spontaneous activity. These parameters were then used for the 3 other columns. The set of four layer 2/3 columns then had to be re-tuned as a whole once introducing lateral excitation and inhibition between columns. We then added the layer 5 neurons and matched their response properties with those seen *in vivo*. Finally, an “update working memory” loop was added to reset the network to a baseline level of activity as has been shown experimentally. After tuning for the optimal responses, responses were then tuned for non-optimal conditions to match experimental evidence showing changes electrophysiological changes with D1 and D2 receptor agonists and antagonists. The time required to tune the network may be expedited by using mean-field calculations, which have recently been developed for two-dimensional models such as the Izhikevich model (Nicola and Campbell, 2013) and by automated parameter tuning frameworks, such as that developed by Carlson et al. (2014).

#### Neuron model

The Izhikevich spiking neuron model was used to govern the dynamics of the spiking neurons in this simulation. The computational efficiency of these point neurons (single compartment) makes them ideal for large-scale simulations. Izhikevich neurons are also highly realistic and are able to reproduce at least 20 different firing modes seen in the brain, which include: spiking, bursting, rebound spikes and bursts, sub threshold oscillations, resonance, spike frequency adaptation, spike threshold variability, and bistability of resting and spiking states (Izhikevich, 2004). Inhibitory and excitatory neurons in the cortex were modeled using the simple Izhikevich model, which are described by the following equations (Izhikevich, 2003):

(1) $$\overset{´}{v}=0.04v^{2}\;+\;5v\;-\;u\;+\;I{\;}^{*}\;\mu_{DA,grp}$$

(2) $$\overset{´}{u}=a\left(bv\;-\;u\right)$$

(3) if v=30,then v=c,u=u+d

where *v* is the membrane potential, *u* is the recovery variable, *I* is the input current, μ is a neuromodulatory factor, and *a, b, c, d* are parameters chosen based on the neuron type. For regular spiking, excitatory neurons, we set *a* = 0.01, *b* = 0.2, *c* = −65.0, *d* = 8.0. For fast-spiking, inhibitory neurons, we set *a* = 0.1, *b* = 0.2, *c* = −65.0, *d* = 2.0. μ is a neuromodulatory factor that is dependent upon the dopamine concentration (*DA*) and neural group *grp*. Neuromodulatory factors are explained in more detail in the Neuromodulation section below.

#### Synapse model

The synaptic input, *I*, driving both excitatory and inhibitory neurons was dictated by simulated AMPA, NMDA, GABA_A_, and GABA_B_ conductances (Izhikevich and Edelman, 2008; Richert et al., 2011). The conductance equations used are well-established and have been described in Dayan and Abbott (2001) and Izhikevich et al. (2004). The total synaptic input to neuron was given by:

(4) $$\begin{array}{l} I=g_{AMPA}\left(v\;-\;0\right)\;+\;g_{NMDA}\frac{{\left[\frac{v\;+\;80}{60}\right]}^{2}}{1\;+\;{\left[\frac{v\;+\;80}{60}\right]}^{2}}\left(v\;-\;0\right)\\ \;+g_{GABA_{A}}\left(v\;+\;70\right)\;+\;g_{GABA_{B}}\left(v\;+\;90\right) \end{array}$$

where *v* is the membrane potential and *g* is the conductance. The conductances change according to the following first order equation:

(5) $${\overset{´}{g}}_{i}\;=\;\frac{-g}{\tau_{i}}$$

where *τ_i_* = 5, 100, 6, 150 ms for *i* = AMPA, NMDA, GABA_A_, GABA_B_ conductances, respectively. When an excitatory (inhibitory) neuron fires, g_AMPA_ and g_NMDA_ (g_GABAA_ and g_GABAB_) increase by the synaptic weight, *wμ_i,DA,conn_*, between pre- and post-synaptic neurons. μ, in this case, is a neurmodulatory factor that is dependent on the conductance (*i*), dopamine concentration (*DA*), and connection (*conn*). Neuromodulatory factors are explained in more detail in the Neuromodulation section below.

### Neuromodulation

Our model incorporated simulated D1 and D2 receptors (Figure 2A). To understand the action of these receptors, it is first important to make clear the distinction between “preferred” and “non-preferred” directions and inputs. A neuron, for example, that shows persistent firing for a cue presented at 180° has a “preferred direction” of 180°. Preferred inputs to these neurons are excitatory inputs that also show persistent firing for a cue presented at 180° (i.e., recurrent excitatory connections, within a column). Non-preferred inputs are excitatory connections from neural groups that have other preferred directions, such as 0 or 90° (i.e., lateral excitatory connections, between columns).

D1 receptors have been shown to be important for blocking non-preferred excitatory inputs to cortical columns in the dlPFC (Arnsten, 2011). D1 receptors mediate the blocking of non-preferred inputs by increasing cAMP levels in spines where non-preferred inputs synapse onto preferred inputs (Vijayraghavan et al., 2007). Thus, when dopamine levels are low in PFC (weakly activating D1 receptors), non-preferred inputs to columns are enhanced. When dopamine levels are optimal, non-preferred inputs are weakened (see Figure 2B). At high levels of dopamine, which may occur during stress, it has been suggested (Arnsten, 2009) that cAMP levels in dendritic spines increase to the point that they weaken all inputs to dlPFC neurons (Figure 2C). Though this is the hypothesized mechanism for selectively gating lateral excitation in the PFC, this mechanism could be implemented in a variety of ways, including presynaptic expression of D1 receptors or D1 receptors expressed on GABAergic neurons (Arnsten, 2011).

We simulated the enhancement of non-preferred inputs when DA levels were low by increasing the strength of lateral excitatory connections onto excitatory neurons (i.e., AMPA and NMDA conductances; see Section Synapse Model) in columns encoding different preferred directions. When DA levels were low, then, μ was set equal to 1.4 for AMPA and NMDA conductances on connections from non-preferred to preferred L3 excitatory connections. When DA levels were optimal, μ was set equal to 1.0 for AMPA and NMDA conductances on connections from non-preferred to preferred L3 excitatory connections. When D1 levels are high, it has been shown that the activity and spatial tuning of dlPFC neurons strongly decreases (Vijayraghavan et al., 2007). This was functionally implemented in our network by decreasing all inputs to L2/3 excitatory neurons of the PFC, as illustrated in Figure 2C. It should be noted that this effect of high D1 receptor stimulation has been shown physiologically, however the exact mechanism is not currently known. We simulated this effect by setting μ equal to 0.8 in equation 1, which decreases the overall input to all excitatory neurons in L2/3 of the PFC. Overall, these neuromodulatory factors were chosen to match experimental data (Vijayraghavan et al., 2007), which suggest low overall activity and spatial tuning degradation in dlPFC with high DA levels and a high overall activity and spatial tuning degradation in dlPFC at low DA levels. It should be noted that this mechanism contrasts with the working model developed by Brunel and Wang (2001), which suggests that D1 receptors mediate persistent activity (recurrent excitatory activity) via activation of NMDA receptors.

The role that D2 receptors play in the PFC, on the other hand, is not as well-understood. D2 receptors, which have been shown to reside exclusively in layer 5 in the PFC (Lidow et al., 1998b), are important for set-shifting (Floresco et al., 2006), and may play a role in cognitive flexibility (Durstewitz and Seamans, 2008) and reward prediction (Gee et al., 2012; Puig and Miller, 2014). The stimulation of D2 receptors has been shown to increase response-related activity of layer 5 neurons in the PFC and a blockade of D2 has suppressed PFC activity. In both cases, there is no effect on delay-related responses (Wang et al., 2004).

This being the case, when D2 receptors were weakly or strongly stimulated in our simulation, we decreased or increased the strength of the excitatory connection, respectively, from the SC to the layer 5 neurons of the PFC. That is, we set μ for AMPA and NMDA conductances (see Section Synapse Model) on SC to layer 5 connections to 0.6 when D2 was low, 1.0 when D2 was optimal, and 1.8 when D2 was high. This had the effect of decreasing and increasing the activity of layer 5 neurons (see Figure 2D) as has been seen experimentally with pharmacological D2 receptor manipulation (Wang et al., 2004). The activity of layer 5 neurons, in turn, affected the clearing of working memory and the inhibition of thalamic inputs onto other cortical columns via the basal ganglia and a non-selective inhibitory pool of neurons (Figure 2A). Specifically, when D2 receptor stimulation was low, weakly activating layer 5 neurons, the inhibitory signal from the inhibitory pool to clear working memory was weak, leading to persistence of working memory activity (see Results). Low D2 receptor stimulation also caused a weak release of inhibition of thalamic inputs to the PFC. When D2 receptor stimulation was high, strongly activating layer 5 neurons, the inhibitory signal to clear working memory was strong. High D2 receptor stimulation also caused a strong release of inhibition of thalamic inputs to the PFC by the BG, allowing noise to leak in to the PFC from the thalamus.

It should be noted that the neuromodulatory changes in our model were implemented to mimic the response properties of neurons as have been shown experimentally with pharmacological manipulations, as opposed to suggesting a synaptic/cellular mechanism. Indeed, though much is known about how dopamine affects cellular and synaptic properties of neurons, our model remains agnostic to this and is focused on how these types of changes in response (firing rate) properties can lead to dysfunction at the circuit level.

### Input presentation

The input to our network was structured according to the oculomotor delayed response (ODR) behavioral paradigm. Each individual experiment can be broken down into four stages: fixation, cue, delay, and response (Figure 3). During the fixation stage, a constant, random Poissonian spike input 5 Hz drove all four columns in the network. When the cue was presented, the inputs to a single column were increased to 35 Hz. This biased drive to that column was removed during the delay period, allowing for recurrent excitatory connections in a column to reverberate and hold onto the cued location in working memory. During the response period, inputs to the MD/SC layer were increased, driving neurons in layer 5 of all columns. This caused layer 5 neurons to clear working memory in layer 2/3 via GABAergic projections from a non-selective inhibitory pool.

## Results

In our results, we first demonstrate that both weak D1 activation and strong D2 activation leads to noise in the PFC by pushing all of the columns into a persistent state and degrading spatial tuning. We then systematically manipulate D1 and D2 receptor stimulation levels from low-to-high in order to see how this changes working memory activity. Non-optimal simulated D1 and D2 receptor levels leads to persistence, noise, and/or low firing rates in our networks. Finally, we look at the behavioral results for the low-to-high receptor stimulation conditions and show that these match well with those seen experimentally in patients with schizophrenia.

### Low D1 and high D2 receptor stimulation levels lead to noise in the PFC

We first examined the responses of the four dlPFC columns of our model when we had weak D1 receptor activation and high D2 receptor activation. Figure 4A shows the firing rates of layer 2/3 neurons in the four columns averaged across multiple runs under optimal conditions (center), weak D1 activation (left), and strong D2 activation (right). When D1 and D2 receptors are optimally activated, noise from excitatory connections between columns is reduced due to D1 receptors. Noise from the thalamus (gated by the BG) is also weak due to controlled activation of layer 5 neurons in the PFC. This optimal condition led to high spatial tuning in working memory (i.e., low noise), as can be seen by the single column (blue trace) dominating during the delay period, and results from a balance between recurrent excitatory inputs within a column and excitatory-inhibitory connections between columns.

When D1 receptors are weakly activated, noise is introduced in the PFC working memory columns (Figure 4A, low D1). This happened as a result of an increase in the excitatory drive between columns (i.e., strengthening non-preferred inputs; Figure 4B). The noise in working memory can be seen in the heightened activity of all columns (poor spatial tuning) during the delay period. We refer to this noise as “internal” noise because it happens as a result of the strengthening of excitatory connections between PFC columns.

When D2 receptors are strongly activated, noise is also introduced into the system (Figure 4A, high D2). In contrast to the weakly activated D1 case described above, the noise when D2 receptors are strongly activated results in the over-activation of layer 5 neurons. Layer 5 neurons released the inhibitory control that basal ganglia neurons have on thalamic inputs that are impinging on PFC layer 2/3 neurons (Figure 4B). This leads to an increase in the overall external excitatory drive to the layer 2/3 neurons in all columns. We refer to this noise as “external” because it happens as a result of an increase in excitatory drive from thalamic neurons.

The result shown in Figure 4A is important because it suggests that weak activation of D1 receptors or over-activation of D2 receptors can lead to noise in the PFC. This is qualitatively consistent with the dynamical systems hypotheses (Durstewitz and Seamans, 2008; Rolls et al., 2008), which suggest that instabilities in persistent firing when D1 receptors are weakly stimulated or D2 receptors are over-stimulated lead to noise in working memory and various symptoms of schizophrenia. Our model, however, is able to reproduce the same results given the more biologically plausible distributions of D1 and D2 receptors. We further suggest that D1 and D2 receptors lead to different “types” of noise, namely, internal noise coming from within the PFC (endogenous noise) and external noise from the thalamus (exogenous noise), respectively.

### Systematic manipulation of D1 and D2 receptor stimulation levels

**Figure 5:**
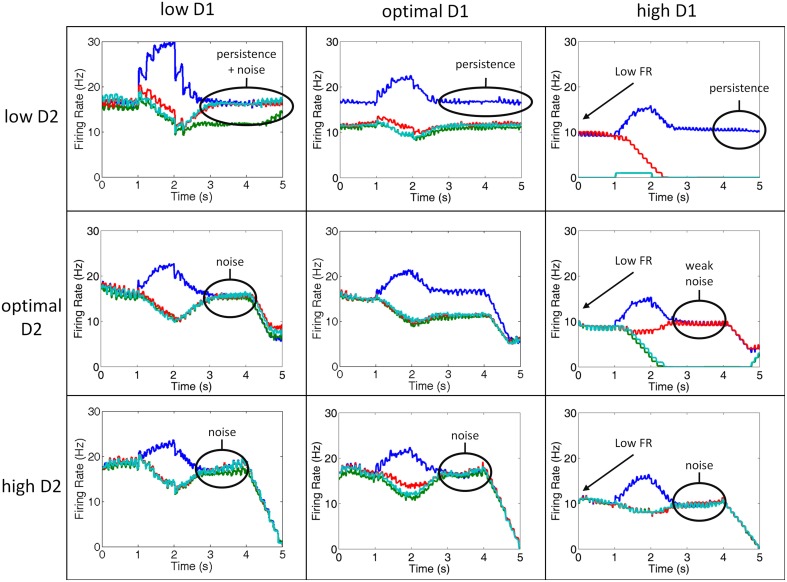
**Simultaneous manipulation of D2 and D1 receptor stimulation from low to high**. Plots showing the firing rates of the layer 2/3 neurons in the 4 separate columns during various conditions of D1 and D2 receptor stimulation for a single, representative trial. Column 1 is shown in blue, column 2 in green, column 3 in red, and column 4 in teal. Low D1 (left column) and high D2 (bottom row) states lead to increases in internal and external noise, respectively. Low D2 (top row) states, on the other hand, leads to perseveration. High D1 (right column) leads to low firing rates and noise.

In order to fully explore the space of possible working memory and behavioral variations that come with D1 and D2 receptor stimulation changes, we simulated low, optimal, and high D1 and D2 receptor stimulation levels. Figure 5 shows a set of 9 graphs for all possible mixtures of low, optimal, and high D1 and D2 receptor activation levels. In each graph, we plot the smoothed firing rate (moving average) of neurons in all four columns for one representative trial of the ODR task (out of the total 50 trials). In all cases, the cue was presented to column 1 (blue) only. In the optimal D1 and D2 case, we see that the model correctly sustains activity only in column 1 over the delay period (Figure 5, center).

We observed several irregularities in working memory that emerge as D1 and D2 receptor activation levels deviate from optimal, including: persistence, noise, and lower firing rates. The lower firing rates occurred as a result of over D1 activation (right column). When D1 was over-activated, the input to all neurons was diminished as explained in the methods. This result, which has been shown experimentally (Williams and Goldman-Rakic, 1995), is thought to be due to an increase in cAMP in the dendrite and is behaviorally correlated with high levels of stress (Vijayraghavan et al., 2007; Arnsten, 2011).

Persistence occurred when D2 receptor activation was low (top column of Figure 5). In our model, when D2 receptors are weakly activated, the activity of layer 5 neurons, which project to a non-selective inhibitory pool of neurons, are correspondingly decreased. This resulted in an inability to clear working memory. Behaviorally, this persistence could lead to perseverative errors or impair attentional set-shifting. In fact, in our model, we found an increase in perseverative errors with low D2 stimulation (see Behavioral Results). Interestingly, a similar result was seen experimentally in rats (Floresco and Magyar, 2006) and humans diagnosed with schizophrenia (Park and Holzman, 1992). Floresco et al. (2006) showed that rats that were given a D2 antagonist in a four-arm radial plus maze task were unable to perform an attentional set-shift. Park and Holzman (1992) discussed in their paper that schizophrenic patients tended to have higher rates of perseverative errors than controls in the ODR task, however, they did not quantify this. It was, however, shown that perseverative errors increase in the Wisconsin card sorting task among patients with schizophrenia (Park, 1997). We show in the behavioral results that perseverative errors are actually higher in the low D1 case, which has also been reported experimentally in rodents (Ragozzino, 2002; Fletcher et al., 2005; Nikiforuk, 2012). Depending on the readout of the motor layer, however, the persistence in working memory could carry over to perseverative errors.

As described above, noise can enter the system via two separate mechanisms in our model: under-activation of D1 receptors or over-activation of D2 receptors. When D1 receptors are under-activated, noise enters the system via excitatory connections between columns in the PFC (internal noise), and when D2 receptors are over-activated, noise enters the system via excitatory connections from the thalamus (external noise). In low D1 (left column) or high D2 (bottom row) states, noise corrupted working memory during the delay period as can be seen by the lack of spatial tuning during the delay period.

### Behavioral results

Changes in D1 and D2 receptor stimulation levels also caused behavioral impairments in our model that were similar to those seen in schizophrenic patients. To simulate a behavioral response, we chose the motor group that had the greatest number of spikes in MOT during the 500 ms before the response occurred. This is reminiscent of accumulator models that have been suggested for decision making and proposed to exist in the brain in areas such as the parietal cortex (Schall et al., 2011). In addition, the overall response of the motor layer had to exceed 6 Hz. If it did not exceed 6 Hz, a random saccade direction was chosen. A random saccade could result in a correct, incorrect, or perseverative response from the model. Perseverative errors were identified as occurring when the same saccade direction was chosen on two consecutive trials. These results were collected over 50 trials and the target position was varied in each trial.

Table 3 shows the percentage of correct saccades and perseverative errors at different D1/D2 receptor stimulation levels. As can be seen in Table 3, as D1 and D2 stimulation levels deviate from the optimal, deficits in behavior occurred, although some deficits were worse than others. Similar behavioral deficits have been shown in the ODR task in patients with schizophrenia (Park and Holzman, 1992; Park, 1997). Specifically, Park and Holzman (1992), Park (1997) found an approximately 12% reduction in correct responses in schizophrenic patients vs. controls in the ODR paradigm as well as in increase in perseverative errors (data not shown in Park and Holzman (1992). We saw, on average, a 32% reduction in the overall number of correct responses and a 5% increase in the number of perseverative errors in the non-optimal conditions. The non-optimal behavioral results will be discussed in more detail below.

**Table 3:** **Percentage of correct and perseverative responses on the ODR task**.

	**Low D1**	**Optimal D1**	**High D1**
**Low D2**	42, 20%	68, 18%	48, 8%
**Optimal D2**	42, 26%	78, 16%	64, 2%
**High D2**	40, 40%	44, 22%	32, 20%

Behavior was the worst when both D1 and D2 receptor activation levels were high. High D1 receptor activation decreased all inputs to a working memory neuron, including lateral excitation, recurrent excitation, and lateral inhibition. The decrease in recurrent and lateral excitation led to low firing rates. The decrease in lateral inhibition, on the other hand, led to less competition between columns. This can be seen in comparing the high D1/high D2 case with the optimal D1/optimal D2 case in Figure 5. Notice in these plots that in between 1 and 2 s (when the cue is presented) there is a stronger dip in the non-preferred columns for the optimal D1/optimal D2 case than the high D1/high D2 case, indicating stronger lateral inhibition. This gets more noise into the system, faster. Behavior, then, was severely impaired due to a faster equivalence in firing rates of all columns (quicker degradation of spatial tuning).

Low D1 receptor stimulation led to behavioral impairments at all levels of D2 receptor stimulation. Low D1 receptor stimulation introduced internal noise into the system by enhancing lateral (between columns) excitatory connections onto excitatory neurons in layer 2/3, It is clear that introduction of noise via lateral excitation should lead to more incorrect responses since all layers will be more likely to drive motor responses. We, however, also found that the number of perseverative errors also increased. This is likely due to the fact that low D1 levels make it so that all cortical columns are activated simultaneously. Simultaneous activation of all columns would lead to them effectively canceling each other out, making it difficult for any one column to “overtake” the previously dominant working memory state and appropriately switch behavioral response. Increases in perseverative errors have also been found with the application of D1 antagonist in rodent set-shifting tasks. These tasks showed a doubling in the number of perseverative errors, similar to our model. These tasks, however, showed increased perseveration with extra-dimensional set-shifting and it is not clear how well this would map to the oculomotor task.

Behavior was also impaired in high D1 conditions when D2 levels were low. When D1 levels are high and D2 levels were low, the overall level of firing did not often exceed the threshold in the motor layer for a decision to be made, thus impairing performance. In fact, 50% of responses were random in the high D1, low D2 condition. Behavior improved when D2 levels were increased to optimal. This resulted from the percentage of random saccades decreasing from 50 to 34%.

Low D2 receptor stimulation also caused behavioral impairments when D1 levels were low or optimal. These impairments resulted from persistent activity in the low D2 state (see Figure 5), which occurred as a result of a weak “resetting” of working memory via projections from layer 5 to the thalamus. The inability to reset to a baseline response level made it difficult for one column to overtake the others when the cue was presented and, therefore, all neurons remained in high firing rate “working-memory” states. Persistent activity also resulted in a slightly higher percentage of perseverative errors in the low and optimal D1 cases. Perseveration with D2 antagonist has been shown in strategy set-shifting experiments in rodents by increasing the number of trials it took them to reach a set number of correct behavioral responses (Floresco et al., 2006). This effect of perseveration was not as strong as was seen in the low D1 case (described above). Behavior improved in the low D2 case as D1 levels increased from low to optimal. The reason for this is clear in moving from the low D1 to optimal D1 case, since lateral excitation decreases, reducing noise. Behavior, however, was impaired in the high D1 case due to overall low overall firing rates.

## Discussion

We developed a spiking neural network model that took into account the differing distributions of D1 and D2 receptors and their effects on synaptic transmission and neuronal activity in order to more accurately characterize how symptoms of schizophrenia may arise. Similar to dynamical systems hypotheses (Loh et al., 2007; Durstewitz and Seamans, 2008), we showed that both low D1 and high D2 states cause an increase in noise in the PFC. This noise, however, is introduced into the cortex via two distinct routes: under-activation of D1 receptors gates in noise between columns in the PFC through excitatory connections and over-activation of D2 receptors gates in noise by driving subcortical areas. We applied this concept in an oculumotor delayed response task where we varied D1 and D2 receptor activation levels simultaneously and saw how this changed both working memory activity and behavior. High D1 states decreased the overall firing rate of neurons and led to the model performing “random” saccadic behavior. Low D1 and/or D2 states, on the other hand, led to perseverative errors as has been seen experimentally in rodents (Ragozzino, 2002; Fletcher et al., 2005; Floresco and Magyar, 2006; Nikiforuk, 2012), with low D1 states leading to the most perseverative errors. Though our simulation modeled a specific behavioral task and brain region, we believe that the similarity in distribution of these receptors across regions in the PFC make the dysfunction highly stereotyped, the variability of which manifests only through differing inputs and outputs to specific regions. Below we explain in more detail how we think this can account for many of the symptoms of schizophrenia.

The low D1/high D2 state of our model is most consistent with experimental data on schizophrenic subjects. Abi-Dargham and colleagues, for example, have shown that D1 receptor occupancy levels were lower in schizophrenic patients than control groups, suggesting weak D1 receptor stimulation (Abi-Dargham et al., 2002). In addition, fMRI BOLD data shows overall greater activation in dlPFC for schizophrenic patients (Manoach et al., 1999), which is consistent with the lowD1/highD2 state for our model (see lower left of Figure 5). The low D1/high D2 state also led to perseverative errors, which have been shown to occur in schizophrenic patients (Park and Holzman, 1992; Park, 1997). Finally, theoretical hypotheses propose the idea that schizophrenic brains are in a lowD1/highD2 state (Loh et al., 2007), and suggest that D2 antagonists combined with D1 agonists may help alleviate many symptoms of schizophrenia.

**Figure 6:**
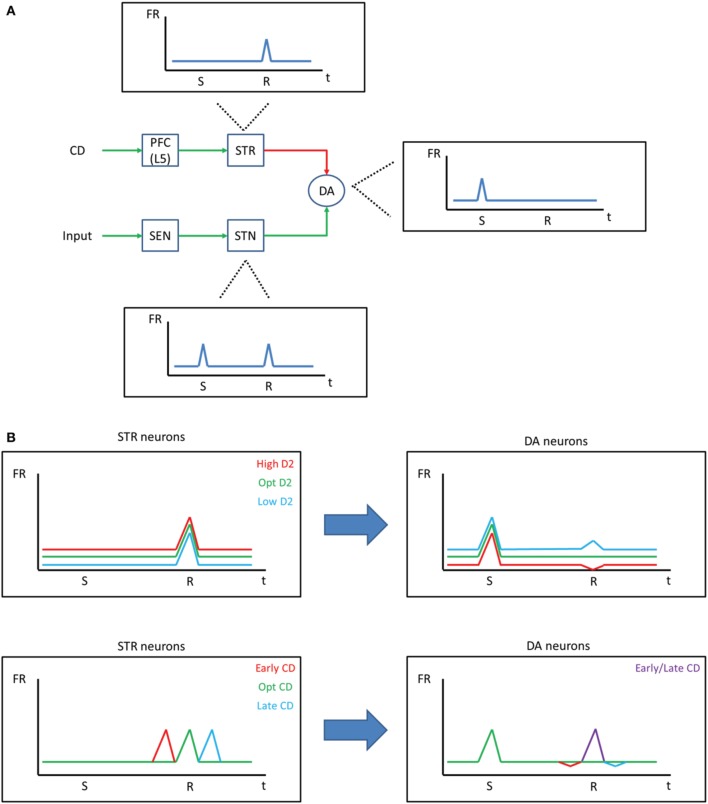
**Negative symptoms in schizophrenia. (A)** Chorley and Seth (2011) developed a model demonstrating how dopamine reward prediction error signals may be learned through the balance of excitatory and inhibitory projections. Excitatory signals from sensory areas fire phasically and drive dopamine neurons during the time of the stimulus (S) and the reward (R). Inhibitory signals from the striatum, on the other hand, also drive dopamine neurons, resulting in a constant firing rate during the time of the reward when the stimulus is predictive of the reward. **(B)** Our model suggests that the D2 state should affect striatum projections to dopamine neurons (top). That is, a high D2 state would increase the strength of inhibition on DA neurons, resulting in an overall lower firing rate for DA neurons and a dip in response at the time of the reward, despite the stimulus being predictive of the reward. Because PFC neurons that project to the striatum are driven by the corollary discharge (CD) in our model, an abnormal corollary discharge, as may be occurring in schizophrenic patients, could ultimately lead to abnormal DA responses (bottom).

The idea that a low firing rate leads to negative symptoms (Loh et al., 2007) conflicts with the high firing rate in the low D1/high D2 state of our model and fMRI data showing an elevated BOLD signal in the PFC of schizophrenic patients (Manoach et al., 1999). We propose, instead, that negative symptoms arise due to noise in the reward prediction error signal. To build upon this idea, we briefly introduce a model (Chorley and Seth, 2011) that is able to account for how reward prediction error signals take shape in midbrain dopamine neurons. In their model, Chorley and Seth suggest that inhibitory signals from the striatum, which are driven by the PFC, must match excitatory signals from subthalamic nucleus for DA neurons to fire phasically for a stimulus predictive of a reward (Figure 6A). Our model predicts that D2 levels would affect the neurons in the PFC that project to the striatum and, therefore, could alter the reward prediction error signal of dopamine neurons. A high D2 state, then, would increase activity in the PFC and lead to overall lower activity in DA neurons and a dip in the DA response at the time of the reward (Figure 6B, top). Our suggested mechanism agrees with recent data that has shown that abnormal prediction errors are associated with negative symptoms in patients with schizophrenia (Moran et al., 2008; Gradin et al., 2011). Negative symptoms would arise, then, as noise in the prediction error computation due to noise in the PFC.

Abnormal corollary discharges are also associated with schizophrenia (Ford et al., 2001; Heinks-Maldonado et al., 2007; Crapse and Sommer, 2008). According to our model, abnormally timed corollary discharge would lead to abnormal reward prediction error signals (Figure 6B, bottom). A similar idea has been proposed by Whitford et al. (2012), who hypothesizes that abnormal myelination in frontal circuits leads to delayed corollary discharges, which ultimately drives the onset of schizophrenic symptoms. Given this circuit, it is possible that the abnormal corollary discharge is, in fact, the source of all of the symptoms of schizophrenia as a result of its changes to the prediction error signal.

One question that arises is: how can D1 and D2 receptor stimulation levels differ? The answer to this may lie in how D1 and D2 receptors respond to tonic and phasic dopamine. It has been suggested that D2 receptors are more responsive to phasic DA signals and D1 receptors are more responsive to tonic levels of DA (Seamans and Yang, 2004). This is likely due to the fact that D2 receptors are located within the synapse and D1 receptors are located extra-synaptically (Winterer and Weinberger, 2004). Looking at this in terms of reward prediction error circuit described above and in Figure 6, it could be then that an abnormal corollary discharge leads to more phasic activity in the VTA (Figure 6B, bottom), which pushes the system into a lowD1/highD2 state.

There are several antipsychotic medications currently available that have been effective in treating positive symptoms of schizophrenia, however, negative and cognitive symptoms still persist (Miyamoto et al., 2012). First generation antipsychotics, including chlorpromazine and haloperidol, are strong D2 antagonists. These medications, however, typically cause movement disorders due to their interaction with dopamine circuits in the basal ganglia. Second and third generation antipsychotics, such as clozapine, have a higher affinity for serotonin receptors (5HT2A) than D2 receptors and, therefore, do not lead to movement-related problems. Interestingly, over-activation of 5HT2A receptors, which are also located in layer 5 of the PFC, has been suggested as a mechanism of action for psychoactive substances that cause hallucinations (Marek and Aghajanian, 1999). This suggests that over-activation of layer 5 neurons in the PFC, in general, could be a mechanism for producing positive symptoms of schizophrenia as well as hallucinations that result from psychoactive drugs. Our model also predicts that cognitive symptoms, which result from D1 mediated increases in excitation between columns, would not be affected by antipsychotic medications since they have a weak affinity for D1 receptors.

The strong correlation between dopaminergic activity and brain disorders has led to many interesting primate and rodent studies that involve manipulation of D1 and D2 receptors in the PFC. D1 receptor levels have been shown to be important for set-shifting in rodents (Ragozzino, 2002; Nikiforuk, 2012) as well as attention and working memory in primates (Arnsten, 2011). It is also interesting to note that a recently developed model of PFC suggests an increase in perseverative errors when external noise was added to the model (Rigotti et al., 2010). Our model similarly shows increases in perseverative errors and working memory impairments at low D1 levels. Furthermore, we predict that perseveration happens as a result of the canceling out of columns that are competing to inhibit the current, dominating, column. Levels of D2 receptor stimulation, on the other hand, alter set-shifting abilities in both humans and rodents (Mehta et al., 2004; Floresco and Magyar, 2006). Specifically, these studies showed that D2 antagonists lead to increases in perseverative errors. We saw only subtle increases in perseverative errors in our model, however, persistence in the firing rate was clear. Therefore, depending on the readout of the motor layer, the persistence in working memory could carry over to perseverative errors.

The computational role of noise in the brain has become an important topic in neuroscience in the past decade. The term stochastic facilitation has been used to label any noise that improves information processing (McDonnell and Ward, 2011; Chakravarthy, 2013; Marro et al., 2013) and is based on the finding in statistical physics that weak signals may be enhanced by noise in non-linear systems (termed “stochastic resonance”). We suggest that D1 and D2 receptors differentially gate noise into the PFC allowing these receptors to play a critical role in information processing and behavior. Adaptively gating internal noise between PFC columns via D1 receptors may broaden tuning curves and allow for more flexible behavior (Arnsten et al., 2012), whereas D2 receptors might also allow for more flexible behaviors (for example, set-shifting) by gating external noise from the thalamus into the PFC. In each case, D1 and D2 receptors can improve behavioral performance by adding flexibility to the behavioral repertoire. Likewise, by reducing the spread of activity, these receptors may be important in situations where quick, decisive action in needed (exploitation). The changes in noise levels due to D1 and D2 receptors stimulation in PFC may also affect encoding and decoding from neural populations in early visual areas by influencing noise correlations and levels of low-frequency oscillations (Cohen and Maunsell, 2009; Mitchell et al., 2009). It will be interesting in the future to investigate how varying spatiotemporal patterns of activity within the PFC, which are likely mediated by D1 and D2 receptors, in turn influence information processing in early visual areas how this is altered in neuropsychiatric disorders.

We discuss a few important aspects of the present model that do not fit empirical data precisely. First, the baseline activity of our model is slightly higher than is seen in experiments. This is likely due to a high background firing rate for the input neurons or lateral excitation strength was a little too high. However, these differences do not have an impact on the overall behavior or the qualitative effect of D1/D2 receptor activation. Our model also predicts a dip in the firing rate of L2/3 neurons when a saccade is initiated. Some neurons in the PFC, however, have a transient spike in activity that coincides with saccade generation and the end of persistent activity (see Figure 1C). This suggests that the clearing of working memory could be caused by a pulse of excitation as shown by Brunel and Wang (2001). There are, however, some neurons in the PFC that do not show this transient spike in activity at the time of saccade generation (Funahashi, 2013). Instead, persistent activity dips (is inhibited) with saccade generation as we see in our model, suggesting the possibility for multiple mechanisms in updating/clearing working memory. Finally, connections between layers 3 and 5 were left out for simplicity sake and because there is less data on the functional connectivity between these two areas. It would be interesting in future models to add these connections to further understand their computational role in working memory.

Our model makes several important predictions. First, we suggest that cognitive symptoms in schizophrenics could arise via the same mechanism as is proposed in patients with ADHD, namely, under-activation of D1 receptors. We propose that the corollary discharge and D2 receptors are important for the gating input into working as well as reward prediction and that these should be preferentially located on subcortical projecting pyramidal neurons in layer 5 as opposed to cortico-cortical neurons (see Shepherd, 2013 for review). Contrary to the dynamical systems hypothesis, our model predicts that the working memory attractor itself is not unstable, rather, improper gating of noise introduces excess “energy” in to the system and unpredictably pushes the network into a persistent state.

Treating schizophrenia, then, may require several adjunctive therapies. D1 and D2 targeting drugs may help to resolve cognitive and positive symptoms, respectively. Our model, however, suggests that any drug that targets receptors on layer 5 neurons in PFC may be appropriate for controlling positive symptoms (similar to second and third generation antipsychotics). Likewise, drugs that enhance working memory but don't specifically act on D1 receptors may be a good option for improving cognitive symptoms. This further suggests that layer and cell-type specific drug therapies may be very important so that we are not influencing other circuits mediated by dopamine, such as the basal ganglia. Our model, in the framework of the model proposed by Chorley and Seth, further suggests that fixing the corollary discharge with remyelination medications (Whitford et al., 2012), for example, may be equally affective and could improve negative symptoms. Because dopamine plays such an important role for learning in the PFC (Otani et al., 1998; Sheynikhovich et al., 2013), it might also be necessary to “reprogram” frontal circuits of schizophrenic patients with therapy and drugs, such as those used to help alleviate phobias (Ressler et al., 2004). This further highlights the importance of early diagnosis and treatment of the disorder. It is our hope that the development of this model will demonstrate the importance of thinking about distributed microcircuits in schizophrenia and other disorders and help to bridge the gap between our understanding of disorders at the cellular and the systems/behavior level.

### Conflict of interest statement

The authors declare that the research was conducted in the absence of any commercial or financial relationships that could be construed as a potential conflict of interest.
